# The Sodium/Proline Transporter PutP of *Helicobacter pylori*

**DOI:** 10.1371/journal.pone.0083576

**Published:** 2013-12-17

**Authors:** Araceli Rivera-Ordaz, Susanne Bracher, Sannia Sarrach, Zheng Li, Lei Shi, Matthias Quick, Daniel Hilger, Rainer Haas, Heinrich Jung

**Affiliations:** 1 Microbiology, Department of Biology I, Ludwig Maximilians University Munich, Martinsried, Germany; 2 Department of Physiology and Biophysics, Weill Cornell Medical College, New York, New York, United States of America; 3 HRH Prince Alwaleed Bin Talal Bin Abdulaziz Alsaud Institute for Computational Biomedicine, Weill Cornell Medical College, New York, New York, United States of America; 4 Center for Molecular Recognition and Department of Psychiatry, Columbia University College of Physicians and Surgeons, New York, New York, United States of America; 5 Max von Pettenkofer Institute for Hygiene and Medical Microbiology, Ludwig Maximilians University Munich, Munich, Germany; University of Cambridge, United Kingdom

## Abstract

*Helicobacter pylori* is cause of chronic gastritis, duodenal ulcer and gastric carcinoma in humans. L-proline is a preferred energy source of the microaerophilic bacterium. Previous analyses revealed that *HpputP* and *HpputA*, the genes that are predicted to play a central role in proline metabolism as they encode for the proline transporter and proline dehydrogenase, respectively, are essential for stomach colonization. Here, the molecular basis of proline transport in *H. pylori* by HpPutP was investigated experimentally for the first time. Measuring radiolabeled substrate transport in *H. pylori* and *E. coli* heterologously expressing *HpputP* as well as in proteoliposomes reconstituted with HpPutP, we demonstrate that the observed proline transport in *H. pylori* is mediated by HpPutP. HpPutP is specific and exhibits a high affinity for L-proline. Notably, L-proline transport is exclusively dependent on Na^+^ as coupling ion, i.e., Na^+^/L-proline symport, reminiscent to the properties of PutP of *E. coli* even though *H. pylori* lives in a more acidic environment. Homology model-based structural comparisons and substitution analyses identified amino acids crucial for function. HpPutP-catalyzed proline uptake was efficiently inhibited by the known proline analogs 3,4-dehydro-D,L-proline and L-azetidine-2-carboxylic acid.

## Introduction

*Helicobacter pylori* is a human pathogen, responsible for type B gastritis and peptic ulcers as well as for increasing the risk of gastric adenocarcinoma and mucosa-associated lymphoid tissue lymphoma of the stomach [1-4]. The bacterium is microaerophilic, exhibits a strict respiratory form of metabolism, and oxidizes organic acids as energy source [5,6]. Amino acids (L-proline, L-serine, L-alanine) are efficiently oxidized by *H. pylori*, and in agreement with their occurrence in human gastric juice, are discussed as main respiratory substrates in the mucus of the stomach [7]. In fact, L-proline is suggested to be the predominant amino acid in the gastric juice of humans infected with *H. pylori* (e.g., 10 mg per g gastric juice), a phenomenon most likely resulting from increased collagen degradation [7-9]. In addition, growth of the bacterium is enhanced by addition of L-proline and L-alanine to modified Eagle’s minimal medium [10]. Importantly, signature-tagged mutagenesis identified a gene predicted to encode a proline transporter (PutP) as one of 47 genes absolutely essential for gastric colonization by *H. pylori* [11]. In addition, degradation of L-proline appears to be important for infection. Inactivation of a gene predicted to encode a proline dehydrogenase (PutA) renders *H. pylori* non-motile and prevents colonization of the stomach of mice [9]. On the contrary, wild-type and a *putA* mutant of the closely related human pathogen *Helicobacter hepaticus* displayed similar levels of infection in mice, but in mice challenged with the *putA* mutant strain, significantly reduced inflammation was observed [12]. Taken together, these observations suggest that L-proline uptake and metabolism are of particular significance for physiology and virulence of *Helicobacter* strains*.*

Experimental evidence for the mechanism of L-proline transport in *H. pylori* is not available yet. Genome analyses predict the existence of minimum three putative L-proline transporters in *H. pylori*: PutP, ProP, and ProVWX (Tomb et al., 1997). The *E. coli* orthologs of these transporters are well characterized. The osmoregulator and secondary transporter ProP and the ABC-type transport system ProVWX transport L-proline and betaines, and are involved in cell adaption to osmotic stress [13-15]. PutP of *E. coli* (EcPutP) is a member of the Na^+^/solute symporter family (SSS family) [16] and catalyzes high affinity uptake of L-proline for subsequent consumption of the amino acid as a source of carbon, nitrogen and energy [17-19]. 

We have set out to obtain experiment-based information on the molecular mechanism of proline uptake into *H. pylori* P12. We found that the strain was able to accumulate external L-proline. Accumulation was dependent on gene *hpp12_0049*, a *putP* ortholog, which proved also to be sufficient for the observed transport activity. Energetic requirements and kinetic properties of the gene product were determined, and presumable inhibitors were tested. The results indicate that gene *hpp12_0049* codes for a high affinity Na^+^/proline symporter. Sequence comparison and homology modeling suggested amino acids potentially involved in ligand binding. The predictions were experimentally tested and led to the identification of amino acids crucial for function. 

## Materials and Methods

### Bacterial strain and plasmids

*E. coli* DH5α [F- ϕ80d *lacZ* ΔM15 Δ(*lacZYA-argF*) U169 *deoR recA1 endA1 hsd* R17(rk-,mk+) *phoA supE44* λ- *thi-1 gyrA96 relA1*] was used as carrier for the plasmids. *E. coli* WG170 (F^-^ *trp lacZ rpsL thi* Δ(*putPA*)*101 proP219*) [20] harboring given plasmids was employed for heterologous expression of *H. pylori* genes and transport assays. Plasmids pT7-5 containing the *lac* promoter/operator [21,22] and pTrc99a [23] were used for all gene manipulations and expression in *E. coli*.

*H. pylori* P12 (originally isolated as 888-0, [24]) was employed as source of gene *hpp12_0049* (*HpputP*), and as homologous expression system. Plasmid pIB6 (Iris Barwig, Lea Holsten and Rainer Haas, unpublished) was used as a shuttle vector for *E. coli* and *H. pylori*, and for constitutive expression of *HpputP* in *H. pylori* from the *alpA* promoter.

### Cultivation conditions

*E. coli* was grown aerobically in Luria-Bertani (LB) medium supplemented with 100 µg ml^-1^ ampicillin when hosting a plasmid at 37°C. 

*H. pylori* P12 was cultured on GC agar plates (Difco) supplemented with 10% horse serum, 10 µg ml^-1^ vancomycin, 1 µg ml^-1^ nystatin and 5 µg ml^-1^ trimethoprim under microaerobic conditions (5% O_2_, 10% CO_2_, 85% N_2_) at 37°C for 2 to 3 days [25]. For liquid cultures, *Brucella* broth (Becton Dickinson) supplemented with 10% horse serum was used with rotary shaking at 100 r.p.m. under microaerobic conditions at 37°C. For selection of *H. pylori* allelic exchange mutants, 10 µg ml^-1^ erythromycin or 10 µg ml^-1^ streptomycin was added. Cells transformed with plasmid pIB6 were maintained in medium supplemented with 8 µg ml^-1^ kanamycin.

### Mutant generation

Genes *hpp12_0049* (*HpputP*) and *hpp12_0050* (*HpputA*) of *H. pylori* P12 were individually or together inactivated by replacement with a *rpsL-erm* cassette using the streptomycin susceptibility counterselection strategy [26]. Briefly, up- and downstream regions of the genes to be replaced were PCR amplified (oligonucleotides #1 – #8, Table S1 in File S1) and cloned into pBluescript II SK+ using restriction sites *Hin*dIII and *Not*I. The *rpsL-erm* cassette was inserted into a *Bam*HI site connecting the cloned up- and downstream regions. Resulting plasmids were used for transformation of *H. pylori* P12, and mutants were selected as described [26,27]. Correct chromosomal insertion of the cassette was verified by PCR fragment analysis using primer pairs binding up- or downstream or within the *rpsL-erm* cassette (oligonucleotides #9 - #17, Table S1 in File S1), and sequencing of the PCR products. 

### Cloning

For heterologous expression in *E. coli*, gene *hpp12_0049* (*HpputP*) was PCR amplified from the genome of *H. pylori* P12 (oligonucleotides #18 and #19, Table S1 in File S1) and cloned into plasmid pT7-5 containing the *lac* promoter and a nucleotide sequence encoding a 6His tag using restriction sites *Nco*I and *Xho*I. The resulting plasmid pT*HpputP6H* contained gene *HpputP* fused at its 3’ end to six His codons. For Western blot analyses, a nucleotide sequence encoding the FLAG epitope (5’- CTGCAGGACTACAAGGACGACGATGACAAG GCCTCGAG-3’ (*Pst*I and *Xho*I sites are underlined) was inserted between 3’ end of *HpputP* and the six His codons using engineered restrictions sites *Pst*I and *Xho*I. The resulting plasmid was named pT*HpputPF6H*. For overexpression of *hpputP*, the gene was cloned into plasmid pTrc99a using restriction sites *Nco*I and *Hin*dIII and yielding plasmid pR*HpputPF6H.*

For complementation of *H. pylori* mutants, *HpputP6H* was PCR amplified from plasmid pT*HpputP6H* (oligonucleotides #20 and #21, Table S1 in File S1) and cloned into the shuttle vector pIB6 using restriction sites *Nde*I and *Not*I yielding plasmid pIB*hpputP6H*.

### Transport of radiolabeled L-proline or Na^+^ into intact cells

*H. pylori* cells were grown in 20 ml liquid medium for 24 h as described above. Subsequently, cells were collected by centrifugation at 3500 r.p.m. at 4°C, resuspended in 50 ml fresh medium, and cultivation was continued for additional 12 h. Cells were harvested by centrifugation, washed with 100 mM Tris-morpholineethanesulfonic acid (Mes) buffer, pH 7.0 containing 150 mM KCl, and resuspended in the same buffer to yield an OD_600_ of 0.8. ^14^C-L-proline uptake was measured at 37°C using 200 µl aliquots of the cell suspension per time point. Transport was measured with ^14^C-L-proline (251 Ci mol^-1^) purchased from American Radiolabeled Chemicals Inc. (St. Louis, USA). It was initiated by simultaneous addition of 50 mM NaCl and 10 µM ^14^C-L-proline (specific radioactivity adjusted to 26 Ci mol^-1^) to an aliquot the cell suspension. After given periods of time, transport was terminated by addition of ice-cold 100 mM KP_i_, pH 6.6/100 mM LiCl and rapid filtration through Durapore 0.45-µm membrane filters (Millipore). Radioactivity of bacteria retained on the filters was quantified by liquid scintillation counting.

^14^C-L-proline uptake into *E. coli* WG170 (PutP^-^A^-^) transformed with either plasmid pT7*HpputP6H*, pR*HpputP6H*, pT7-5, or pTrc99a was determined as previously described [28]. Uptake of 20 µM [^22^Na^+^]Cl (86 Ci mol^-1^, American Radiolabeled Chemicals, Inc.) was performed with *E. coli* WG170 harboring either pT7*HpputP6H* or pT7-5 (as control) in the presence or absence of 200 µM L-proline in 100 mM Tris/Mes, pH 6.0 and 20 µM 5-(*N,N*-hexamethylene) amiloride at 25°C. Reactions were quenched with ice-cold 100 mM KP_i_, pH 6.0/100 mM LiCl and rapid filtration through 0.75 µm GF/F filters (Advantec MFS, Inc.). 

### Purification of HpPutP

*HpputP* was overexpressed in *E. coli* WG170 transformed with plasmid pR*HpputP6H*. Cells were grown as described above, and expression was initiated by addition of 0.5 mM isopropyl thio-β-D-galactoside (IPTG) when the culture reached an OD_420_ of 1.0. Cultivation was continued for 3 h. Cells were harvested by centrifugation, washed with 100 mM KP_i_, pH 7.5/2 mM β-mercaptoethanol (BME) and resuspended in the same buffer. Inverted membrane vesicles were prepared by passage of the cell suspension through a high-pressure cell disruptor (Constant Systems Ltd.) followed by low speed centrifugation at 12,000 g for 30 min at 4°C to remove unbroken cells. Membranes were collected by centrifugation at 230,000 g for 90 min at 4°C, and washed with 100 mM KP_i_, pH 7.5/2 mM BME. Finally, membranes were resuspended in 100 mM KP_i_, pH 8.0/2 mM BME/10% glycerol (w/v)/10 mM imidazole/300 mM NaCl to yield a protein concentration of 5 mg ml^-1^. *n*-Dodecyl-ß-D-maltopyranoside (DDM) was added stepwise to a final concentration of 1.5% (w/v) while stirring at 4°C. After additional stirring for 30 min the sample was centrifuged at 230,000 g for 20 min. The resulting supernatant was incubated with Ni^2+^-NTA (1 ml resin pre-equilibrated with 50 bed volumes of 100 mM KP_i_, pH 8.0/2 mM BME/10% glycerol (w/v)/10 mM imidazole/300 mM NaCl/0.04% DDM (w/v) (buffer E) for 45 min with gentle shaking at 4°C. The protein-resin complex was then packed into a column, and unbound protein was removed by washing with 50 bed volumes of buffer E. Subsequently, the resin was washed with 16 bed volumes of buffer E containing 30 mM imidazole. HpPutP6H was eluted from the column with 200 mM imidazole in buffer E. 

### Reconstitution of HpPutP

Preformed liposomes were prepared and destabilized with 0.12% Triton X100 as described [29]. Detergent destabilized liposomes were mixed with purified protein in a 100:1 ratio (w/w) and incubated at room temperature under gentle agitation for 10 min. Detergent was removed by adding Bio-Beads SM-2 prepared according to [30] at a wet weight bead/detergent ratio of 10:1 and 5:1 (w/w) for DDM and Triton X100, respectively. After 1 h of incubation at room temperature fresh Bio-Beads were added and incubation was continued for an additional hour. After the third addition of Bio-Beads incubation was continued over night at 4°C. Bio-Beads were removed by filtration on glass silk, and the turbid proteoliposome suspension was dialyzed three times against 100 mM KP_i_ pH 7.5/2 mM BME at 4°C. Proteoliposomes were concentrated by centrifugation at 300,000 g for 90 min, frozen in liquid N_2_ and stored at -80°C.

### Transport of L-proline into proteoliposomes

The transport assay was adapted from [29]. Proteoliposomes reconstituted with HpPutP6H were diluted in 100 mM KP_i_, pH 7.5/2 mM BME/5 mM MgCl_2_ to yield a protein concentration of ~0.1 mg ml^-1^, and extruded through a 400 nm filter at room temperature. Proteoliposomes were collected by centrifugation and resuspended in the above buffer at a protein concentration of ~1 mg ml^-1^. ^14^C-L-proline uptake was started by a 200-fold dilution of 1 µl aliquots of the proteoliposome suspension into desired buffers containing 10 μM ^14^C-L-proline (26 Ci/mol) and 0.2 μM valinomycin. Depending on the driving force to be established, the following buffers were applied: Na^+^ free 100 mM Tris/Mes, pH 7.5/2 mM BME/5 mM MgCl_2_ with 50 NaCl (*smf*) or 50 mM LiCl (*lmf*) or no further additions (electrical potential); Na^+^ free 100 mM Tris/Mes, pH 6.0/2 mM BME/5 mM MgCl_2_ (*pmf*) or 100 mM KP_i_, pH 7.5/2 mM BME/5 mM MgCl_2_ (facilitated diffusion, control). Transport assays were terminated at a given time by quenching of the reaction with 3 ml ice cold 100 mM KP_i_, pH 6.6/100 mM LiCl and immediate filtration using Millipore filters type GSTF 02500, 0.2 μm pore size.

### Determination of Na^+^

Na^+^ concentrations in buffers used for transport assays were determined with a VARIAN AA240 atomic absorption spectrometer.

### Protein determination

Determination of protein was performed according to a modified Lowry method [31] for total membrane protein, according to Bradford [32] for detergent solubilized protein, and by the amido black method [33] for protein in proteoliposomes.

### Western blotting

Relative amounts of HpPutP with given amino acid replacements in membranes of *E. coli* WG170 and *H. pylori* P12 were estimated by Western blot analysis with HRP-linked mouse anti-Penta His antibody (Santa Cruz Biotechnology Inc., Santa Cruz, USA) or anti-FLAG antibody (Sigma-Aldrich, St. Louis, USA) directed against the 6His tag and FLAG epitope, respectively, at the C terminus of HpPutP similar as described before [34].

### Homology modeling

A homology model of HpPutP was built with Modeller 9v2 [35], using the crystal structure of vSGLT (PDB: 3DH4) as the template. The placements of the bound substrate L-proline and Na^+^ ion were adjusted according to the results of our previous modeling and simulation studies of EcPutP [36].

## Results

### Proline uptake into *H. pylori*

**Figure 1:**
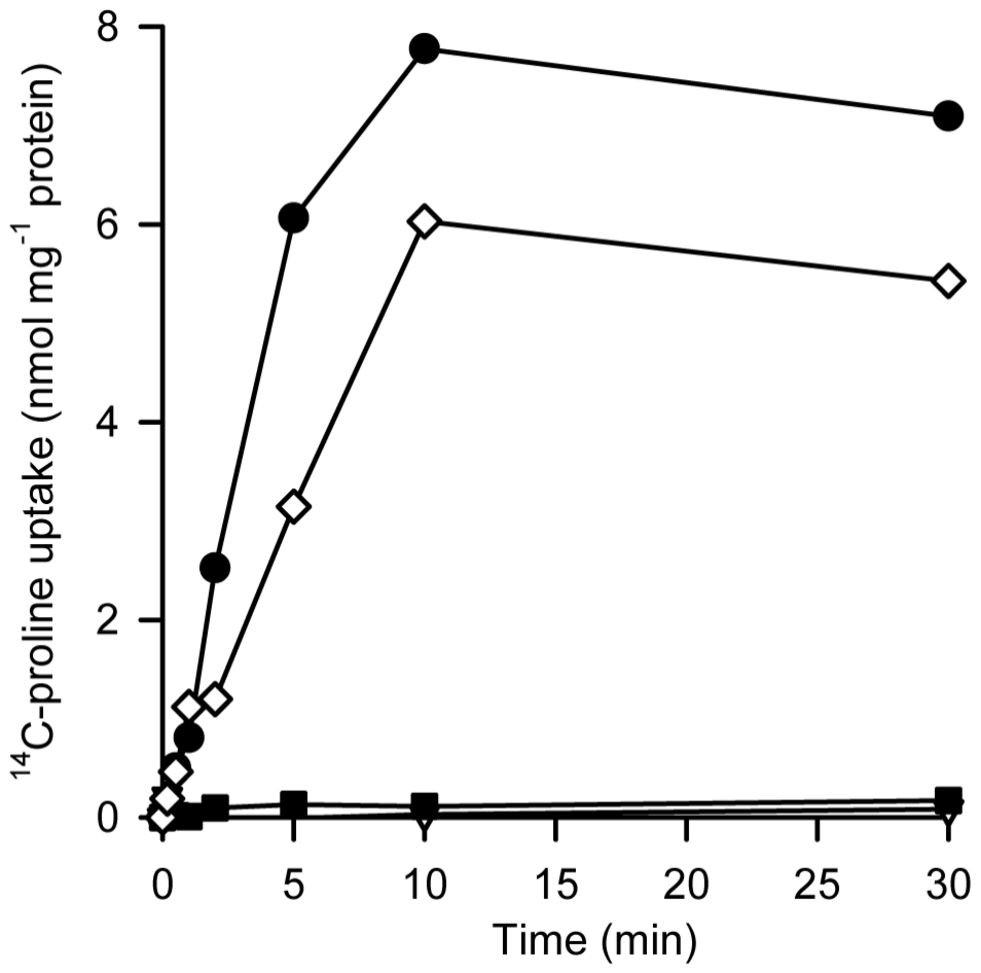
Time course of ^14^C-L-proline uptake into *H*. ***pylori* P12**. Cells were grown and prepared as described in *Experimental* *Procedures*. For the transport assay, 200 µl aliquots of a cell suspension (OD_600_=0.8 in 100 mM Tris/Mes, pH 7.0/150 mM KCl) were prepared per time point. Transport was initiated by simultaneous addition of 50 mM NaCl and 10 µM ^14^C-L-proline (26 Ci mol^-1^) (final concentrations). After given periods of time at 37°C, transport was terminated by addition of ice cold 100 mM KP_i_, pH 6.6/100 mM LiCl and rapid filtration. Radioactivity of bacteria retained on the filters was quantified by liquid scintillation counting. (closed circles) wild type; (open diamonds) *HpputP* mutant plus pIB*HpputP6H*; (closed squares) *HpputPA* mutant; (open triangles) *HpputP* mutant. The data points represent the average of two parallel measurements. Three repeats of the experiment with independently grown and treated cells yielded similar relationships between *H*. *pylori* wild type, mutants and control with activities varying by a factor of up to three between the individual experiments.

*H. pylori* P12 was able to take up ^14^C-L-proline after growth in complex medium and suspension in transport buffer (Figure 1). Analysis of the genome of the strain (NC_011498 [37]) identified *hpp12_0049* as a gene encoding a putative proline transporter. An alignment of the deduced amino acid sequence revealed 50% identity with the Na^+^/proline symporter PutP of *E. coli* (EcPutP) (Figure S1 in File S1). Furthermore, secondary structure analyses predicted 13 transmembrane helices (TMs) exactly as described for EcPutP (Figure S2 in File S1). Deletion of gene *hpp12_0049* (further referred to as *HpputP*) from the genome of strain P12 inhibited proline uptake to values below the detection limit. The transport phenotype of the *H. pylori* mutant was complemented by expression of *HpputP* from plasmid pIB*hpputP6H* (Figure 1). Growth of *H. pylori* in complex medium (Brucella broth) was only slightly inhibited by deletion of *HpputP*, *HpputA*, or *HpputPA* (Figure S3 in File S1).

For a more detailed analysis of L-proline transport kinetics of *H. pylori* P12, uptake was determined at varying L-proline concentrations in transport buffer supplemented with 50 mM NaCl. Plotting of the initial uptake rates versus increasing L-proline concentrations led to a hyperbolic saturation curve (Figure S4A in File S1). The Michaelis-Menten parameter *k*_*m*(*Pro*)_ was 19.4±6.9 µM, while *V*_*max*_ varied between 2 and 6 nmol min^-1^ mg^-1^ in independent experiments, likely due to differences in the energy status of the cells. Varying the NaCl concentration at a constant ^14^C-L-proline concentration of 10 µM revealed a stimulation of proline uptake by NaCl (Figure S4B in File S1). The NaCl concentration causing half maximum stimulation of L-proline uptake (*k*_*0.5*_(Na+_*)*_) was determined to be 1.0±0.5 mM. Note, however, that without addition of NaCl, the extracellular Na^+^ concentration of the cell suspension used for transport was between 70 to 120 µM. Attempts to further reduce the extracellular Na^+^ concentration by repeated washing of cells with Na^+^-free transport buffer inhibited transport irreversibly. Also pre-incubation with the respiratory substrate serine did not stimulate transport.

The results indicate that the product of gene *hpp12_0049* (*HpputP*) is responsible for the observed L-proline uptake in *H. pylori* P12; and that other potential proline transport systems predicted by genome analyses (ProP, ProU) or unknown mechanisms did not play a significant role in L-proline uptake under the test conditions. Furthermore, the data lend first support for the prediction that HpPutP functions as a Na^+^/proline symporter.

### Characterization of HpPutP in *E. coli*

**Figure 2:**
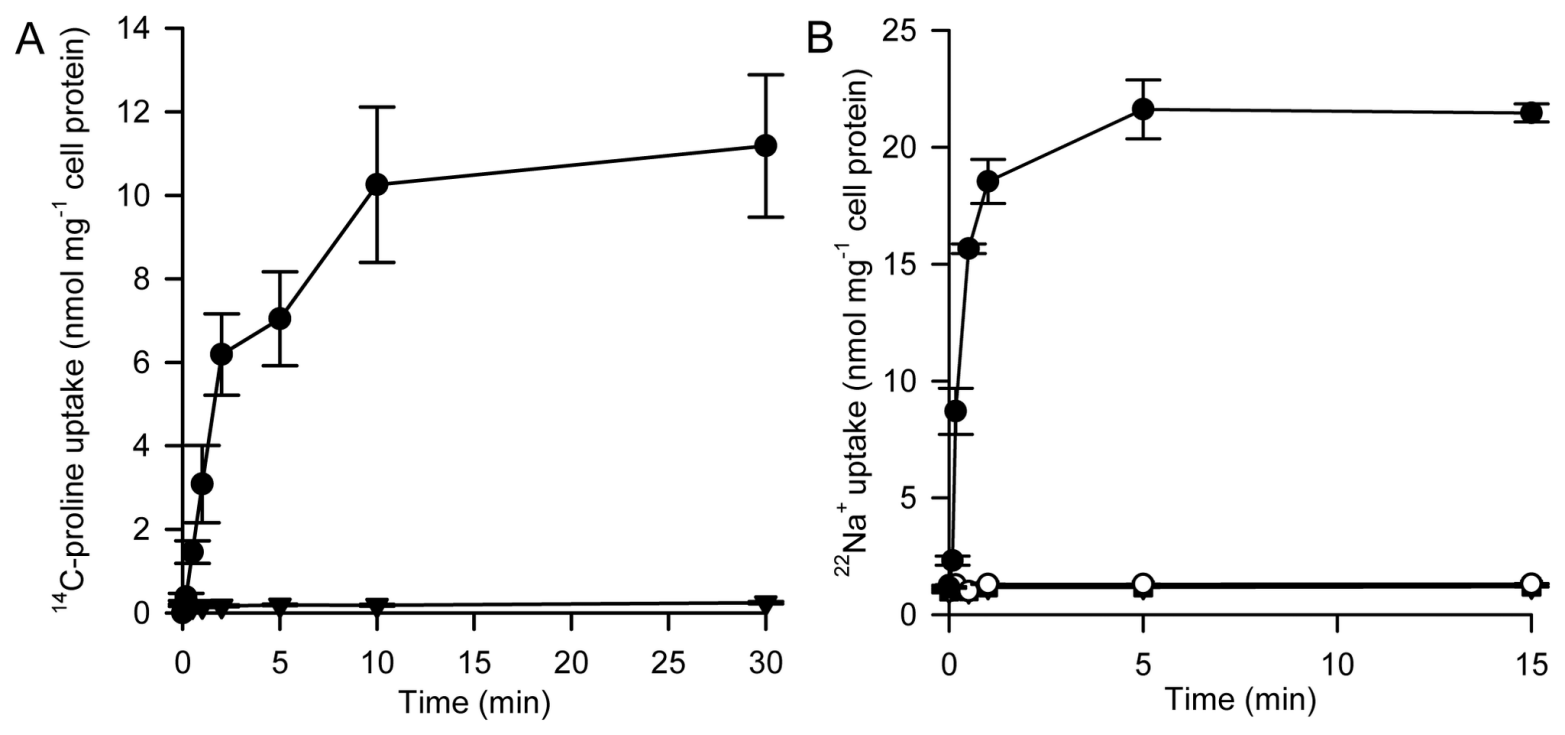
Activity of HpPutP in *E. coli* WG170 (PutP^-^A^-^). (*A*) Uptake of 10 µM ^14^C-L-proline (26 Ci mol^-1^) into *E*. *coli* WG170 harboring HpPutP was assayed in the presence of 50 mM NaCl and 20 mM D-lactate (Na^+^-salt) as electron donor at 25°C as described [28]. (closed circle) cells harboring HpPutP; (closed triangle) cells transformed with pT7-5 (= negative control). (*B*) Uptake of 20 µM ^22^Na^+^ (86 Ci mol^-1^) into *E*. *coli* WG170 harboring HpPutP (circles) or plasmid pT7-5 without *HpputP* (squares) were analyzed in the presence (closed symbols) and absence (open symbols) of 200 µM L-proline. Data are shown as mean ± SEM of triplicate determinations.

Expression of *HpputP* in *E. coli* WG170 (PutP^-^A^-^), a strain that has been routinely used for the characterization of EcPutP, complemented the transport-negative phenotype of the strain (Figure 2A). Furthermore, an inwardly directed concentration gradient of L-proline caused accumulation of ^22^Na^+^ in cells demonstrating coupling of Na^+^ and L-proline transport by HpPutP (Figure 2B). Analysis of initial transport rates at varying L-proline concentrations in transport buffer supplemented with 50 mM NaCl yielded a *k*_*m*(*Pro*)_ value of 1.8±0.2 µM. *V*_*max*_ correlated with the transporter amount in the membrane and reached values of 1.9±0.5 nmol min^-1^ mg^-1^ and 35.6±1.2 nmol min^-1^ mg^-1^ when expression proceeded from *lac* and *trc* promoters, respectively (Figure S5 in File S1). Variation of the Na^+^ concentration at constant ^14^C-L-proline (10 µM) yielded a *k*_*0.5(Na+)*_ value of 17.0±0.9 µM. 

Since the kinetic parameters of the *H. pylori* transporter heterologously expressed in *E. coli* are similar to the ones of the *E. coli* ortholog, our data further foster the conclusion that HpPutP functions as a Na^+^/proline symporter. However, the kinetic parameters of HpPutP in *E. coli* differ to some extent from the parameters obtained in *H. pylori*. The latter phenomenon may be explained by differences of the composition of the cytoplasmic membranes of both bacteria.

### Energetics of HpPutP-catalyzed transport in proteoliposomes

To unequivocally assess whether HpPutP was exclusively responsible for the observed L-proline transport activity in our cellular test systems and to explore its energetic characteristics of transport, HpPutP was solubilized from *E. coli* membranes, purified by Ni^2+^-NTA affinity chromatography and reconstituted into proteoliposomes (Figure S6 in File S1), a membraneous test system devoid of native membrane proteins that could potentially interfere with the characterization of HpPutP. HpPutP-containing proteoliposomes were loaded with 100 mM KPi (pH 7.5), and ^14^C-L-proline accumulation was tested as function of various inwardly directed presumable driving forces [sodium motive force (smf), lithium motive force (lmf), proton motive force (pmf), or membrane potential (*Δψ*)] by creating an outward-directed K^+^ diffusion gradient in the presence of valinomycin, and changing the ionic composition and/or pH of the transport buffer. 

**Figure 3:**
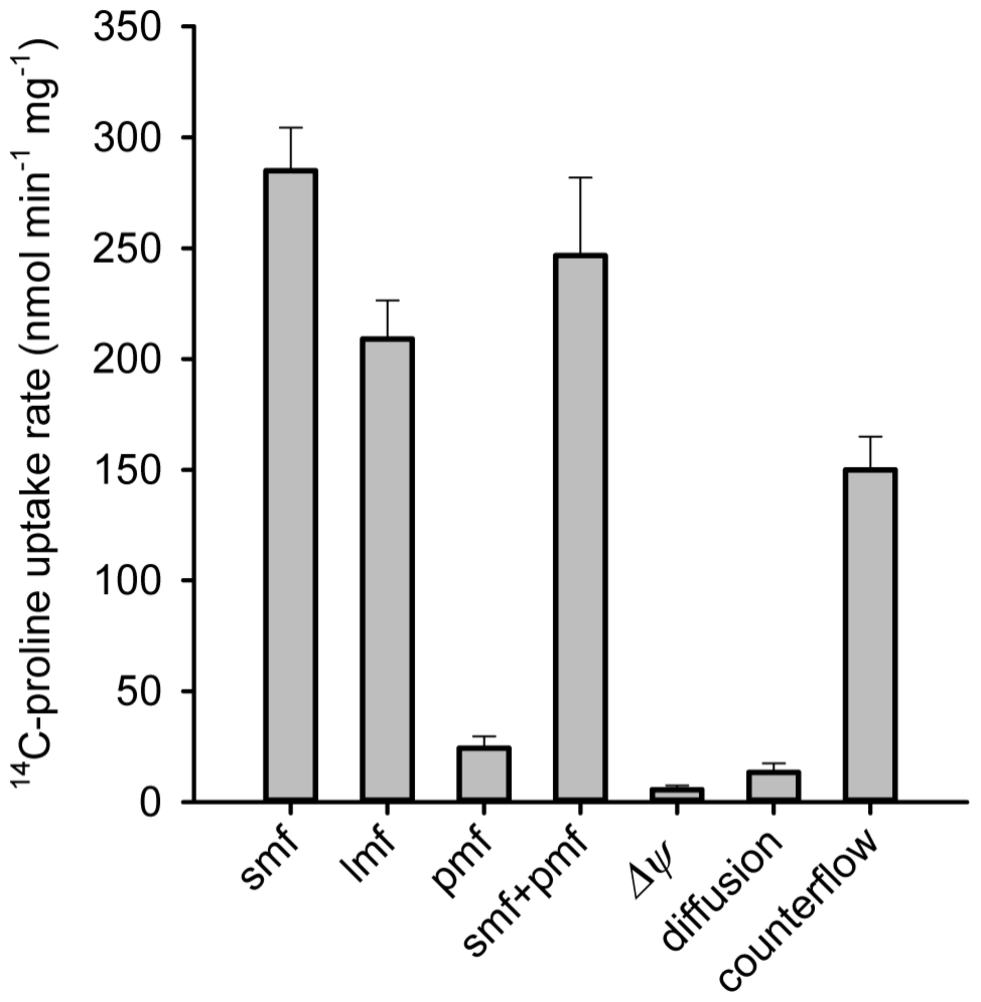
Energetics of ^14^C-L-proline uptake into proteoliposomes containing purified HpPutP. Proteoliposomes in 100 mM KP_i_, pH 7.5 (about 1 mg HpPutP ml^-1^) were diluted 200fold into 100 mM Tris/Mes, pH 7.5 containing 2 mM β-mercaptoethanol, 5 mM MgSO_4_, 0.2 μM valinomycin, 10 μM ^14^C-L-proline (26 Ci mol^-1^) and 50 mM NaCl (smf), or LiCl (lmf), or no further additions (*Δ*ψ). In addition, proteoliposomes were diluted into 100 mM Tris/Mes, pH 6.0 containing 0.2 μM valinomycin (pmf), or 100 mM KP_i_, pH 7.5 (diffusion). Proteoliposomes preloaded with 10 mM L-proline were diluted 200-fold into 100 mM KP_i_, pH 7.5 containing ^14^C-L-proline (256 Ci mol^-1^) (counterflow). Transport was assessed with a rapid filtration method as described in *Experimental* *Procedures*, and data are shown as mean ± SEM of triplicate determinations.

Imposition of a *smf* led to the highest initial uptake rates (up to 300 nmol min^-1^ mg^-1^) under the test conditions confirming Na^+^ as a coupling ion (Figure 3). Also a *lmf* proved to be an efficient driving force indicating that Na^+^ could be substituted by Li^+^. In contrast, a *pmf* was neither able to drive uphill transport of proline nor stimulated *smf*-driven proline transport suggesting that H^+^ did not work as a coupling ion in HpPutP-catalyzed transport although the native environment of *H. pylori* is relatively acidic. Similarly, *Δ*ψ alone did not cause accumulation of ^14^C-L-proline in proteoliposomes. Finally, in the absence of other driving forces, an outwardly directed concentration gradient of L-proline stimulated the uptake of external ^14^C-L-proline in proteoliposomes based on the counterflow mechanism (Figure 3). 

### Structure-function relationships in HpPutP

**Figure 4:**
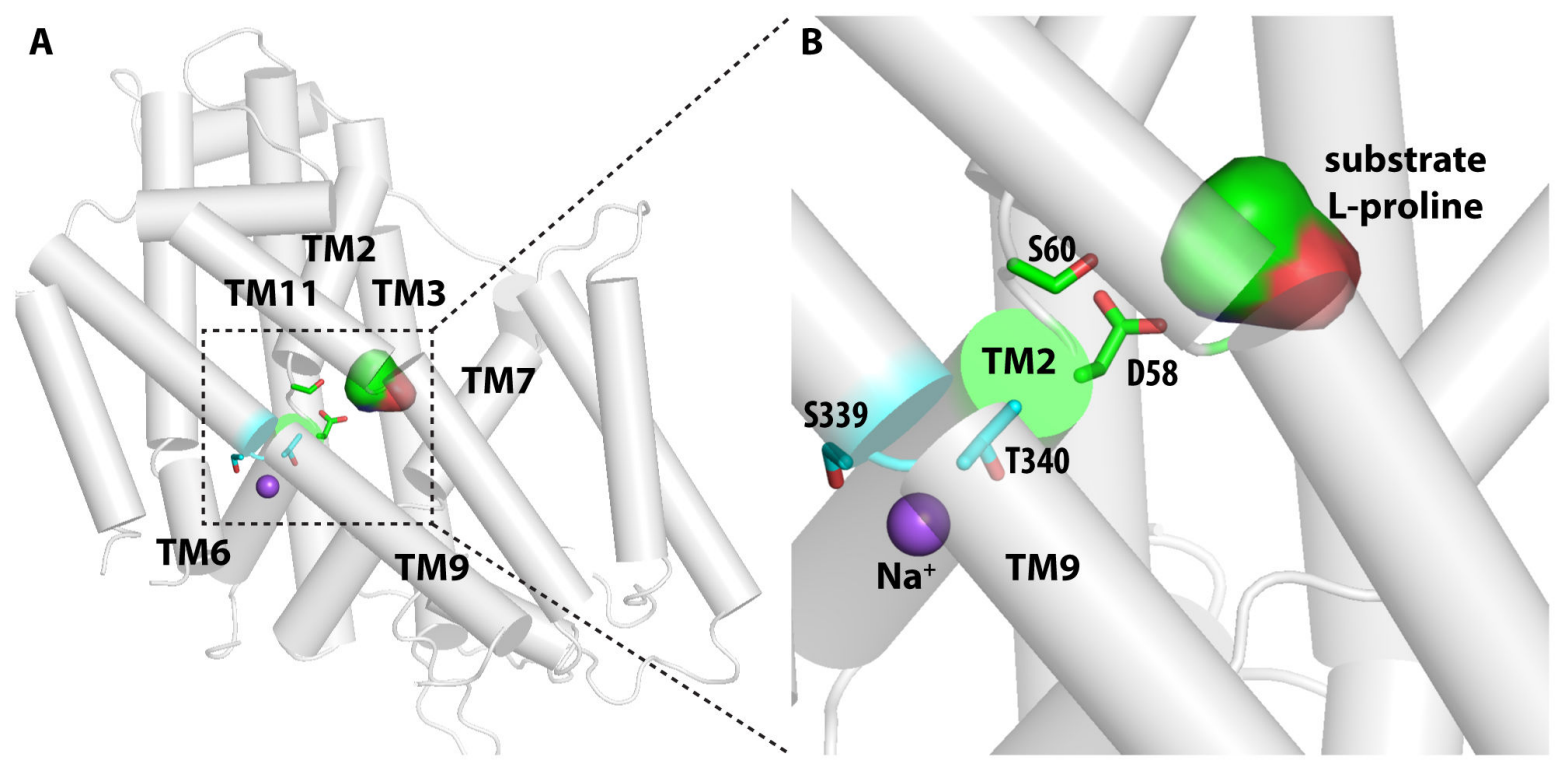
Homology model of HpPutP. (*A*) Overview of the homology model of HpPutP. The substrate binding site is enclosed by TMs 2, 3, 7, and 11, while the bound Na^+^ ion is coordinated by residues from TMs 2, 6, and 9. (*B*) The zoom-in view of the predicted Na^+^ and L-proline binding sites. The substrate and Na^+^ binding residues that have been mutated (see text) are shown in stick representation.

**Figure 5:**
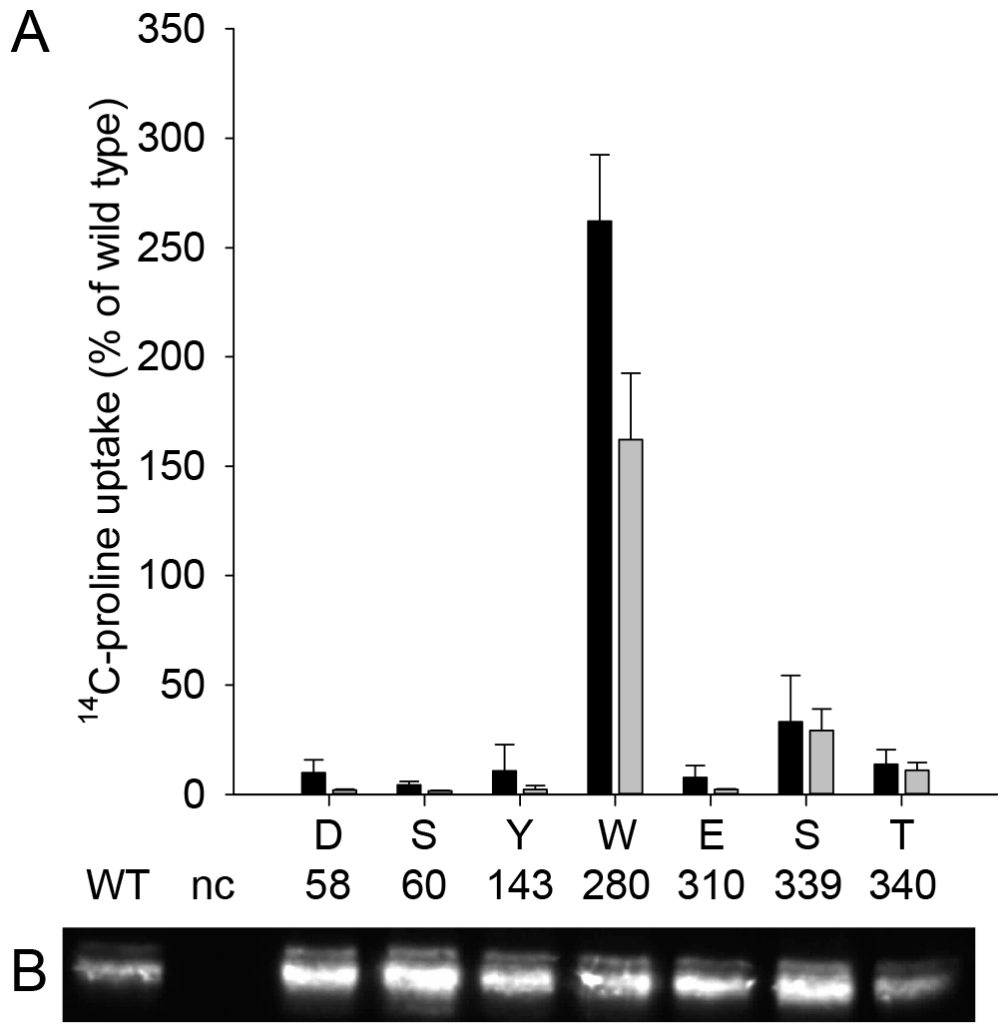
Effect of the placement of cysteine at given amino acid positions on HpPutP in *E. coli* WG170 (PutP^-^A^-^). (*A*) Initial rates of uptake (black columns) and maximum levels of accumulation (grey columns) of 10 µM ^14^C-L-proline (26 Ci mol^-1^) in *E*. *coli* WG170 harboring HpPutP with given substitutions were determined by transport measurements as described in the legend of *Figure 2A*. (*B*) Relative amounts of HpPutP with given amino acid replacements in membranes of *E*. *coli* WG170 were estimated by Western blot analysis with HRP-linked anti-FLAG IgG directed against the FLAG epitope at the C terminus of HpPutP similar as described before [34].

Sequence comparisons identified amino acids in HpPutP that are conserved in L-proline specific members of the SSS family (Asp58, Ser60, Tyr143, Trp280) or in these and other members of the SSS family (Glu310, Ser339, Thr340). Based on our HpPutP homology model (see *Experimental Procedures*), Asp58 and Ser60 are involved in substrate binding, while Ser339 and Thr340 are coordinated to Na^+^ (Figure 4). To explore the significance of these amino acids for HpPutP function, Cys was individually placed at these positions and transport activity was determined. Cys in place of Asp58, Ser60, Tyr143, and Glu310 caused a drop of transport below the detection limit (Figure 5A). Activities of HpPutP-S339C and HpPutP-T340C were severely reduced (5 to 15 % of the wild type). On the contrary, substitution of Trp280 by Cys led to an almost twofold stimulation of the uptake rate. A Western blot analysis revealed that all HpPutP variants were present in the membrane in amounts comparable to the wild type (Figure 5B). Therefore, the observed differences in transport activity were attributed to defects in the transport cycle and were not due to defects in gene expression, membrane insertion, or protein stability.

### Inhibitors of HpPutP

**Figure 6:**
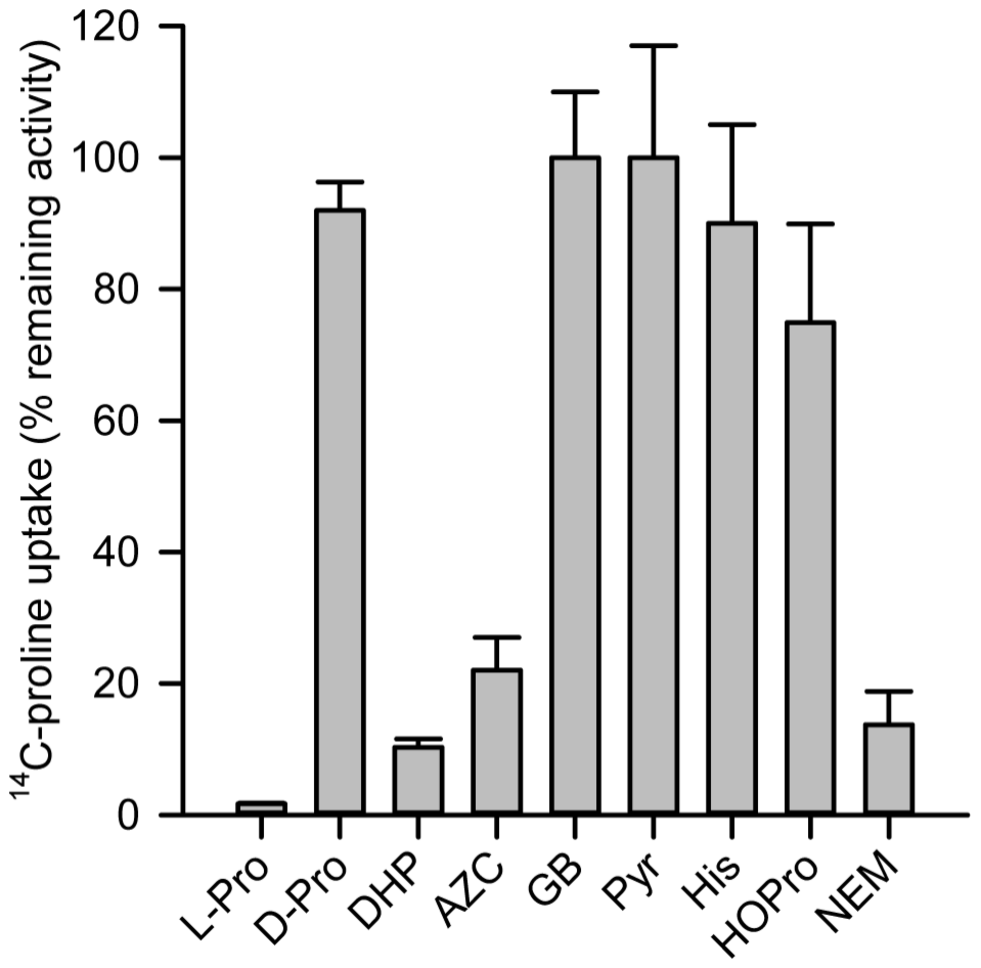
Screen for inhibitors of HpPutP activity in *E. coli* WG170 (PutP^-^A^-^). Initial rates of uptake of 10 µM ^14^C-L-proline (10 Ci mol^-1^) in *E*. *coli* WG170 harboring HpPutP with given substitutions were determined by transport measurements as described in the legend of *Figure 2A*. Putative inhibitors were added in 100fold molar access. NEM was added to the cells suspension at a concentration of 2 mM and incubated for 10 min prior to the start of ^14^C-L-proline uptake. Initial rates of triplicate determinations (shown as mean ± SEM) are represented as percentage of the rate in the absence of inhibitor. (L-Pro) L-proline; (D-Pro) D-proline; (DHP) 3,4-dehydro-D,L-proline; (AZC) L-azetidine-2-carboxylic acid; (GB) glycine betaine; (Pyr) pyrollidine; (His) histidine; (HOPro) hydroxy proline; (NEM) N-ethyl maleimide.

In order to explore the substrate specificity of HpPutP, different compounds structurally related to L-proline were tested for the ability to inhibit L-proline uptake (Figure 6). 3,4-dehydro-D,L-proline (DHP, double bound in ring) and L-azetidine-2-carboxylic acid (AZC, four atom instead of a five atom ring) proved as efficient inhibitors of L-proline uptake as previously shown for PutP of *E. coli* and *Salmonella* [19,38]. Dixon Plot analysis revealed that inhibition was competitive with *k*_*i*_ values of 5.7±0.9 µM (DHP) and 44.2±6.3 µM (AZC) (Figure S7 in File S1). On the contrary, D-proline, pyrollidine, hydroxyproline, histidine, and glycine betaine did not significantly affect L-proline transport when used in up to 100fold molar access over L-proline (Figure 6). Furthermore, HpPutP was inhibited by N-ethyl maleimide probably by modification of minimum one of the three cysteine residues of the transporter. The results suggested that HpPutP is an enantioselective transporter specific for L-proline. The ring structure and size as well as the carboxyl group are important for binding. 

## Discussion

Based on the previously shown crucial role of the proline metabolism in *H. pylori* stomach colonization [9,11], we have investigated the functional properties of the predicted proline transporter HpPutP, product of gene *hpp12_0049*. We demonstrated that i) *H. pylori* P12 is able to transport L-proline, ii) transport of L-proline in cells grown in Brucella medium solely depends on *hpp12_0049*, iii) the isolated gene product, HpPutP, is sufficient to drive proline accumulation, iv) HpPutP is specific for L-proline, v) the *smf* is the driving force for transport, and vi) L-proline transport is obligatory coupled to the flux of Na^+^. We conclude that HpPutP is a Na^+^/L-proline symporter. 

How does HpPutP compare to the well-characterized Na^+^/L-proline symporter of *E. coli*? Expression of *HpputP* complements the transport negative phenotype of an *EcputP* mutant. The kinetic parameters *k*_*m*(*Pro*)_ and *k*_*0.5(Na+)*_ of HpPutP in *E. coli* are similar to the values determined for EcPutP and in agreement with high affinities of the transporter for both L-proline and Na^+^. The significantly lower transport rates of HpPutP compared to EcPutP can be attributed in large part to differences in the amount of protein in the cytoplasmic membrane (Figure S5 in File S1). In *H. pylori*, HpPutP-catalyzed L-proline uptake is stimulated by Na^+^ suggesting that the transporter functions as a Na^+^/L-proline symporter also in its native environment. However, the kinetic parameters *k*_*m*(*Pro*)_ and *k*_*0.5(Na+)*_ of HpPutP in *H. pylori* differ by about one and two orders of magnitude, respectively, from the parameters measured in *E. coli*. This observation may be explained by differences in membrane composition affecting the catalytic cycle of HpPutP. For example, differing from *E. coli* the cytoplasmic membrane of *H. pylori* contains relatively high levels of lyso-phosphatidyl–ethanolamine and cholesterol [39,40]. Note, that analyses of the Na^+^ dependence were hampered by the fact that extensive washing of *H. pylori* irreversibly renders the cells transport-inactive preventing analyses of Na^+^-dependent transport in the lower micromolar range. In any case, the determined kinetic parameters of HpPutP in *H. pylori* fit well to the physiological conditions in the stomach. Biopsies of the stomach of infected persons contain NaCl and L-proline at concentrations of 49±6 mM and 125±39 µM, respectively [7,41]. Such an environment provides saturating conditions with regard to the Na^+^ and L-proline concentrations for HpPutP function. 

A Cys substitution analysis of seven conserved amino acids identified six amino acids (Asp58, Ser60 in TM2, Tyr143 in TM4, Glu310 in eL4’, Ser339, Thr340 in TM9) as particular important for HpPutP function. Asp58 and Ser60 are located in the immediate vicinity of the predicted L-proline binding site, and side-chain alterations at these positions may hamper binding of the substrate. The equivalent amino acids in EcPutP are also of particular functional significance and were originally proposed to participate in Na^+^ and L-proline binding, respectively [34,42]. Alternatively, the residues may be involved in coupling ion and substrate transport by transmitting conformational alterations between binding sites [43]. Furthermore, alignment of the model with the crystal structures of vSGLT and LeuT suggests that Ser339 and Thr340 constitute part of a Na^+^ binding site in HpPutP. The site corresponds to Na^+^ site 2 (Na2) in LeuT [44], and is conserved within the SSS. Replacement of the amino acids at equivalent sites in EcPutP, vSGLT, and the human Na^+^/I^-^ symporter alters transport kinetics and apparent Na^+^ affinities dramatically [43,45-47]. Contrary to Ser339 and Thr340, Tyr143 is conserved only within eubacterial and archaeal members of the SSS family with experimentally demonstrated or predicted proline specificity, but are replaced by other amino acids in SSS members of different specificity (e.g., PanF, vSGLT, SGLT1). In EcPutP, replacement of the corresponding amino acid (Tyr140) also alters transport kinetics and decreases the apparent proline affinity about 10fold [43]. Therefore, Tyr143 of HpPutP is suggested to play a role in proline binding. The idea is further supported by the observation that tyrosine at the position corresponding to that of Tyr143 is part of the amino acid binding pockets of the non-homologous transporters LeuT (Y108) and ApcT (Y97) [44,48]. Finally, the complete inhibition of HpPutP activity upon substitution of Glu310 is unexpected. The amino acid is located in the periplasmic loop connecting TMDs 8 and 9 (7’ and 8’ in the 10 TMD core structure). In LeuT and the Na^+^/benzylhydantoin transporter Mhp1 (cation/nucleobase transporter family) this loop presumably participates in conformational alterations associated with the transport cycle [49,50]. We speculate that the loop functions as a gate controlling access of ligand binding sites in the middle of the transporters, and that in the case of HpPutP tertiary interactions of Glu310 are essential for the gating mechanism.

In an attempt to identify structural features required for L-proline recognition by HpPutP, we assayed the effect of various compounds on the kinetics of L-proline transport. DHP and AZC proved as efficient competitive inhibitors of transport suggesting that 4- and 5-membered rings fit into the substrate binding pocket as previously shown for PutP of *E. coli* and *Salmonella* [19,38]. The carboxylate seems to be essential for binding since pyrrolidine does not affect uptake. Compounds with a substitution at the ring (hydroxyproline) or other ring structures (histidine) appear to be excluded from binding. 

Taken together, these results may facilitate the design of new synthetic inhibitors of HpPutP activity that can effectively block L-proline uptake in *H. pylori*.

## Supporting Information

File S1 **Table S1, Figures S1-S7.** Table S1. Oligonucleotides. Figure S1. Alignment of the amino acid sequences of PutP of *E. coli* and *H. pylori* and SGLT of *V. parahaemolyticus* (vSGLT). The alignment was performed with the complete amino acid sequences of the transporters using CLUSTAL OMEGA followed by manual adjustment. Figure S2. Prediction of transmembrane helices in PutP of *H. pylori*. The analysis was performed with the TMHMM Server 2.0. Figure S3. Effect of the deletion of *put* genes on growth of *H. pylori*. Cells were grown in Brucella broth under microaerophilic conditions as described in *Experimental* *Procedures*. The optical density was determined at 600 nm at given time points. Average values and standard deviations were calculated from three parallel measurements. The entire experiment was independently repeated four times yielding similar results. Figure S4. Kinetics of ^14^C-L-proline uptake into *H. pylori*. Cells were grown in Brucella broth under microaerophilic conditions as described in *Experimental* *Procedures*. For the transport assay, 200 µl aliquots of a cell suspension (OD_600_=0.8 in 100 mM Tris/MES, pH 7.0/150 mM KCl) were prepared per time point. Initial rates of ^14^C-L-proline uptake were determined (*A*) at ^14^C-L-proline concentrations varying from 0.5 µM to 250 µM in the presence of 50 mM NaCl, and (*B*) at a constant ^14^C-L-proline concentration of 10 µM and NaCl added at concentrations varying from 0.07 mM to 250 mM using the rapid filtration assay. Data points represent the mean of duplicate determinations of a representative experiment. Three repeats of the experiment with independently grown and treated cells yielded similar *k*_*m*(*Pro*)_ and *k*_*o.5(Na+)*_ values with maximum activities varying by a factor of up to three between the individual experiments. Figure S5. Comparison of the amounts of EcPutP and HpPutP in membranes of *E. coli* WG170. Expression of *EcputP* (Ec) and *HpputP* (Hp) was achieved form the promoters *lac* (plasmids pT*HpputPF6H*, pT*EcputPF6H*) and *trc* (plasmids pR*HpputPF6H*, pR*EcputPF6H*). Plasmids pT7-5 and pTrc99a served as negative controls (nc). Relative amounts of the transporters were estimated by Western-Blot analysis with HRP-linked anti-FLAG IgG directed against the FLAG epitope at the C termini of EcPutP and HpPutP. Figure S6. Purification and reconstitution of HpPutP. Cells of *E. coli* WG170 transformed with plasmid pR*HpputP6H* were grown and membranes were prepared as described in *Experimental* *Procedures*. The protein (5 mg ml^-1^ total membrane protein) was solubilized with 1.5 % n-dodecyl-ß-D-maltopyranoside (DDM) and purified by Ni^2+^-NTA affinity chromatography. Out of 45 mg total membrane protein applied to 1 ml Ni^2+^-NTA agarose about 1 mg of HpPutP with a purity of about 95 % was obtained. (*A*) SDS-PAGE and Coomassie stain, (*B*) Western Blot of individual steps of the purification procedure. (*C*) Scheme of the reconstitution procedure. Liposomes were preformed from an *E. coli* polar lipid extract, detergent destabilized, incubated with purified HpPutP at a lipid to protein ratio of 100 to 1 (w/w), and proteoliposomes were formed by stepwise removal of the detergent with Bio-Beads SM2. Figure S7. Dixon plot analysis of the inhibition of ^14^C-L-proline uptake by (*A*) 3,4-dehydro-D,L-proline (DHP) and (*B*) L-azetidine-2-carboxylic acid (AZC). Uptake of ^14^C-L-proline (26 Ci mol^-1^) into *E. coli* WG170 harboring HpPutP was assayed in the presence of 50 mM NaCl and 20 mM D-lactate (Na^+^-salt) at the indicated inhibitor concentrations at 25°C.
(PDF)
