# Supplementary material for: The Sodium/Proline Transporter PutP of Helicobacter pylori
Source: PLoS One. 2013 Dec 17;8(12):e83576. doi: 10.1371/journal.pone.0083576 (PMC3866251; doi:10.1371/journal.pone.0083576)
Supplement: File S1 — Table S1, Figures S1-S7. Table S1. Oligonucleotides. Figure S1. Alignment of the amino acid sequences of PutP of E. coli and H. pylori and SGLT of V. parahaemolyticus (vSGLT). The alignment was performed with the complete amino acid sequences of the transporters using CLUSTAL OMEGA followed by manual adjustment. Figure S2. Prediction of transmembrane helices in PutP of H. pylori. The analysis was performed with the TMHMM Server 2.0. Figure S3. Effect of the deletion of put genes on growth of H. pylori. Cells were grown in Brucella broth under microaerophilic conditions as described in Experimental Procedures. The optical density was determined at 600 nm at given time points. Average values and standard deviations were calculated from three parallel measurements. The entire experiment was independently repeated four times yielding similar results. Figure S4. Kinetics of 14C-L-proline uptake into H. pylori. Cells were grown in Brucella broth under microaerophilic conditions as described in Experimental Procedures. For the transport assay, 200 µl aliquots of a cell suspension (OD600=0.8 in 100 mM Tris/MES, pH 7.0/150 mM KCl) were prepared per time point. Initial rates of 14C-L-proline uptake were determined (A) at 14C-L-proline concentrations varying from 0.5 µM to 250 µM in the presence of 50 mM NaCl, and (B) at a constant 14C-L-proline concentration of 10 µM and NaCl added at concentrations varying from 0.07 mM to 250 mM using the rapid filtration assay. Data points represent the mean of duplicate determinations of a representative experiment. Three repeats of the experiment with independently grown and treated cells yielded similar k m(Pro) and k o.5(Na+) values with maximum activities varying by a factor of up to three between the individual experiments. Figure S5. Comparison of the amounts of EcPutP and HpPutP in membranes of E. coli WG170. Expression of EcputP (Ec) and HpputP (Hp) was achieved form the promoters lac (plasmids pTHpputPF6H, pTEcputPF6H) and trc (pl [file pone.0083576.s001.pdf]

Supporting Information

The sodium/proline transporter PutP of *Helicobacter pylori*

Araceli Rivera-Ordaz,[1] Susanne Bracher,[1] Sannia Imrich,[1] Zheng Li,[2] Lei Shi,[2,3] Matthias Quick,[4] Daniel Hilger,[1] Rainer Haas,[5] and Heinrich Jung[1*]

[1]Microbiology, Department of Biology I, Ludwig Maximilians University Munich, Martinsried, Germany.

[2]Department of Physiology and Biophysics, Weill Cornell Medical College, New York USA.

[3]HRH Prince Alwaleed Bin Talal Bin Abdulaziz Alsaud Institute for Computational Biomedicine, Weill Cornell Medical College, New York, USA.

[4]Center for Molecular Recognition and Department of Psychiatry, Columbia University College of Physicians and Surgeons, New York, USA.

[5]Max von Pettenkofer Institute for Hygiene and Medical Microbiology, Ludwig Maximilians University Munich, Munich, Germany.

Table S1. Oligonucleotides. Restriction sites used for cloning are underlined.

No.	Name	Sequence (5' to 3')	Application
#1	Del*putP*_As	CTATCATCTAAGCTTTGGATCGTGTTTTG	*HpputP* replacement, PCR upstream region of *HpputP*
#2	Del*putP*_Aas	CCACAAGTCAGGATCCGCTGAAAAATAA	*HpputP* replacement, PCR upstream region of *HpputP*
#3	Del*putP*_Bs	AACAATAGGGGATCCTAAAACAACATG	*HpputP* replacement, PCR downstream region of *HpputP*
#4	Del*putP*_Bas	GCGATTTCAGCGGCCGCACTGAGCTTTTTAC	*HpputP* replacement, PCR downstream region of *HpputP*
#5	Del*putA*_As	GGGTCTTCTAAGCTTAAGTCAAATTTA	*HpputA* replacement, PCR upstream region of *HpputA*
#6	Del*putA*_Aas	CCACAAGTCAGGATCCGCTGAAAAATAA	*HpputA* replacement, PCR upstream region of *HpputA*
#7	Del*putA*_Bs	TGAATCGTCGGATCCTTTTTGCATGAG	*HpputA* replacement, PCR downstream region of *HpputA*
#8	Del*putA*_Bas	TTATCTTTTGCGGCCGCTAGAGCGGTGTT	*HpputA* replacement, PCR downstream region of *HpputA*
#9	*putP*check1	ATTCTTGTAATTCTAAATC	check *HpputP* replacement
#10	*putP*check2	AAAATTACGAAATCCCGGC	check *HpputP* replacement
#12	*putA*check3	CGCCGCTAAAATCGCGCTC	check *HpputA* replacement
#13	*putA*check4	TGCTGAGATTGTTCTAACG	check *HpputA* replacement
#14	*erm*scheck5	CATAAGTACGGATATAATACG	check gene replacement
#15	*Erm*ascheck6	CGTATTATATCCGTACTTATG	check gene replacement
#16	*rpsL*scheck7	TTTGAAGTGATCAGCTATATC	check gene replacement
#17	*rpsL*ascheck8	ATAGCTGATCACTTCAAAGCC	check gene replacement
#18	pT*HpputP*_s	AGGAGATCACCATGGGACATGTTG	cloning of *HpputP* into pT7-5
#19	pT*HpputP*_as	CTCATTTGAGCTCGAGGCATGCTTCAAGCTC	cloning of *HpputP* into pT7-5
#20	pB*HpputP*_s	AGGAAAGGACATATGGGACATGTTG	cloning of *HpputP* into pIB6
#21	pB*HpputP*_as	GCTTATCATGCGGCCGCTTAGTGGTGGTGGTGGTGGTGGCT	cloning of *HpputP* into pIB6

```
HpPutP      MGHVVL--STPIVTMFVVYSLLMLYIGFYFYK----QNETTEDYFLGDRSMGPVISALSA
EcPutP      ---MAI--STPMLVTFCVYIFGMILIGFIAWR----STKNFDDYILGGRSLGPFVTALSA
vSGLT       MSNIEHGLSFIDIMVFAIYVAIIIGVGLWVSRDKKGTQKSTEDYFLAGKSLPWWAVGASL
               :    *   :  * :*   :: :*:   :      :. :**:*. :*:     . *
                 TM2                        TM3
HpPutP      GASDMSGWLLMGLPGALYVGGLINSHIAIGLSLGALINWVFVAKRLRIYTSVIANSITIS
EcPutP      GASDMSGWLLMGLPGAVFLSGISESWIAIGLTLGAWINWKLVAGRLRVHTEYNNNALTLP
vSGLT       IAANISAEQFIGMSGSGYSIGLAIASYEWMSAITLIIVGKYFLPIF-I--EKG--IYTIP
             *:::*.  ::*: *: :  *:  .      ::.. *     .  . :         *:
                                       TM4                         TM5
HpPutP      DYFETRFSDDKHILRLISAFVILIFFIFYISSGLVSGAKLFEATFGIQYTYALSIGTLII
EcPutP      DYFTGRFEDKSRILRIISALVILLFFTIYCASGIVAGARLFESTFGMSYETALWAGAAAT
vSGLT       EFVEKRF--NKKLKTILAVFWISLYIFVNLTSVLYLGGLALETILGIPLMYSILGLALFA
            ::.  **  . :   :::.: * ::: .  :* :  *.  :*: :*:    ::   :
                                 TM6
HpPutP      VSYTFLGGYKAVCWTDLIQGLLMMSALIVVPIVMIIHLGGIGE---GIKIIREIKPENLS
EpPutP      ILYTFIGGFLAVSWTDTVQASLMIFALILTPVIVIISVGGFGD---SLEVIKQKSIENVD
vSGLT       LVYSIYGGLSAVVWTDVIQVFFLVLGGFMTTYMAVSFIGGTDGWFAGVSKMVDAAPGHFE
            : *:: **  ** *** :*  ::: . ::.  : :  :**      .:. : :    ...
                                       TM7
HpPutP      FLQ-----------GSSVVAIISSLAWGLGYFG-QPHILVRFMSIRSIRDVPKATTIGIS
EcPutP      MLK-----------GLNFVAIISLMGWGLGYFG-QPHILARFMAADSHHSIVHARRISMT
vSGLT       MILDQSNPQYMNLPGIA-VLIGGLWVANLYYWGFNQYIIQRTLAAKSVSEAQKGIVFAAF
            ::            *   *         * *.* :  *: * :.  *  .  :.  :.
             TM8
HpPutP      WMVISLIGACVMGLLGVAYVH--KFD-------L----SLEDPEKIFIVMSQLLFNPWIT
EcPutP      WMILCLAGAVAVGFFGIAYFN--DHP-------ALAGAVNQNAERVFIELAQILFNPWIA
vSGLT       LKLIVPFLVVLPGIAAYVITSDPQLMASLGDIAATNLPSAANADKAYPWLTQFL-PVGVK
              .:    :  :*: . :                       : :: :  ::*:*    :
                       TM9                                    TM10
HpPutP      GILLSAILAAVMSTASSQLLVSSSTIAEDFYATIFNKNAPQKLVMVISRLSVLGVACIAF
EcPutP      GILLSAILAAVMSTLSCQLLVCSSAITEDLYKAFLRKHASQKELVWVGRVMVLVVALVAI
vSGLT       GVVFAALAAAIVSSLASMLNSTATIFTMDIYKEYISPDSGDHKLVNVGRTAAVVALIIAC
            *::::*: **::*: :. *   :: :: *:*   :  .: :: :: :.*  .: .  :*
                                 TM11
HpPutP      FISTDR--NASILSIVSYAWAGFGASFGSVILFSLFWSRMTRIGAIAGMLSGASTVILYD
EcPutP      ALAANP--ENRVLGLVSYAWAGFGAAFGPVVLFSVMWSRMTRNGALAGMIIGALTVIVWK
vSGLT       LIAPMLGGIGQAFQYIQEYTGLVSPGILAVFLLGLFWKKTTSKGAIIGVVASIPFALFLK
             ::         .  :.     .  ..  *.*:.::*.: *  **: *:: .   .:. .

HpPutP      KFG--KSFLDIYEIVPGFIVASAAIVVFSLFSSVRAGTKEAFETMLKEIESLKH------
EcPutP      QFG----WLGLYEIIPGFIFGSIGIVVFSLLGKAPSAAM---QKRFAEADAHYHSAPPSR
vSGLT       FMPLSMPFMDQ--MLYTLLFTMVVI-AFTSL-----------STSINDDDPKGISVTSSM
             :     ::    ::  ::.    * .*: :           .. : : :

HpPutP      --------------------------
EcPutP      LQES----------------------
vSGLT       FVTDRSFNIAAYGIMIVLAVLYTLFW
```

Figure S1. Alignment of the amino acid sequences of PutP of *E. coli* and *H. pylori* and SGLT of *V. parahaemolyticus* (vSGLT). The alignment was performed with the complete amino acid sequences of the transporters using CLUSTAL OMEGA [1] followed by manual adjustment. The location of the TMs of the 10 helix core (TMs 2 to 11 of vSGLT and PutP) is highlighted in yellow. TM boundaries are assigned based on the structure of vSGLT [2]. (red) apolar; (green) polar; (blue) negatively charged (purple) positively charged amino acids.

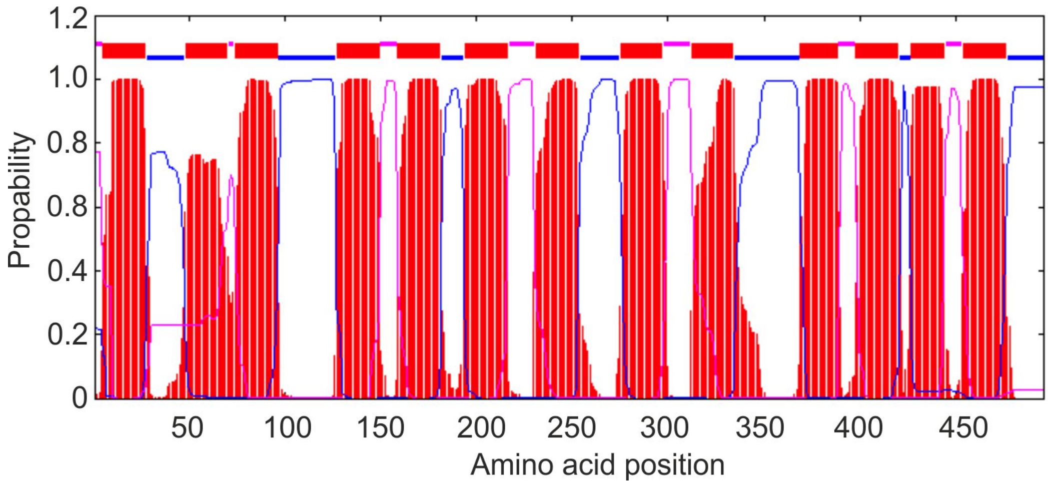

Figure S2. Prediction of transmembrane helices in PutP of *H. pylori*. The analysis was performed with the TMHMM Server 2.0 [3]. (red) transmembrane domain, (blue) inside, (pink) outside).

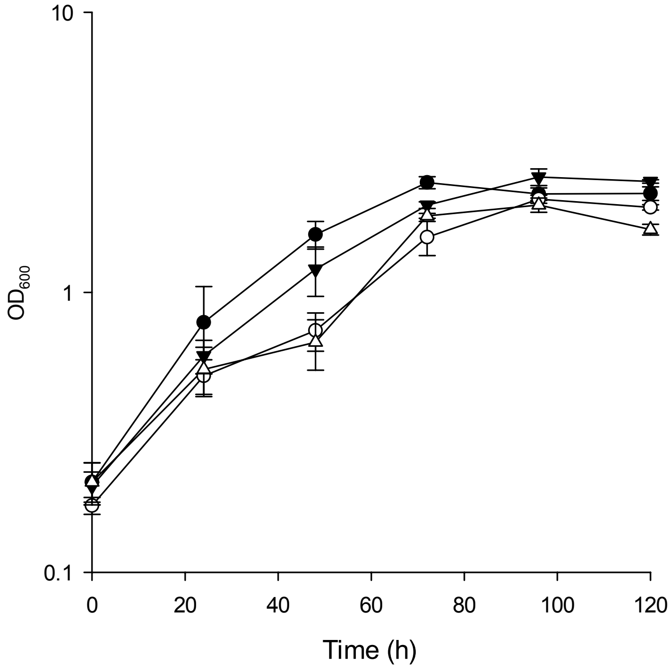

Figure S3. Effect of the deletion of *put* genes on growth of *H. pylori*. Cells were grown in Brucella broth under microaerophilic conditions as described in *Experimental Procedures.* The optical density was determined at 600 nm at given time points. Average values and standard deviations were calculated from three parallel measurements. The entire experiment was independently repeated four times yielding similar results. (closed circles) wild type; (open circles) *putP* deletion mutant; (closed triangles) *putA* deletion mutant; (open triangles) *putPA* deletion mutant.

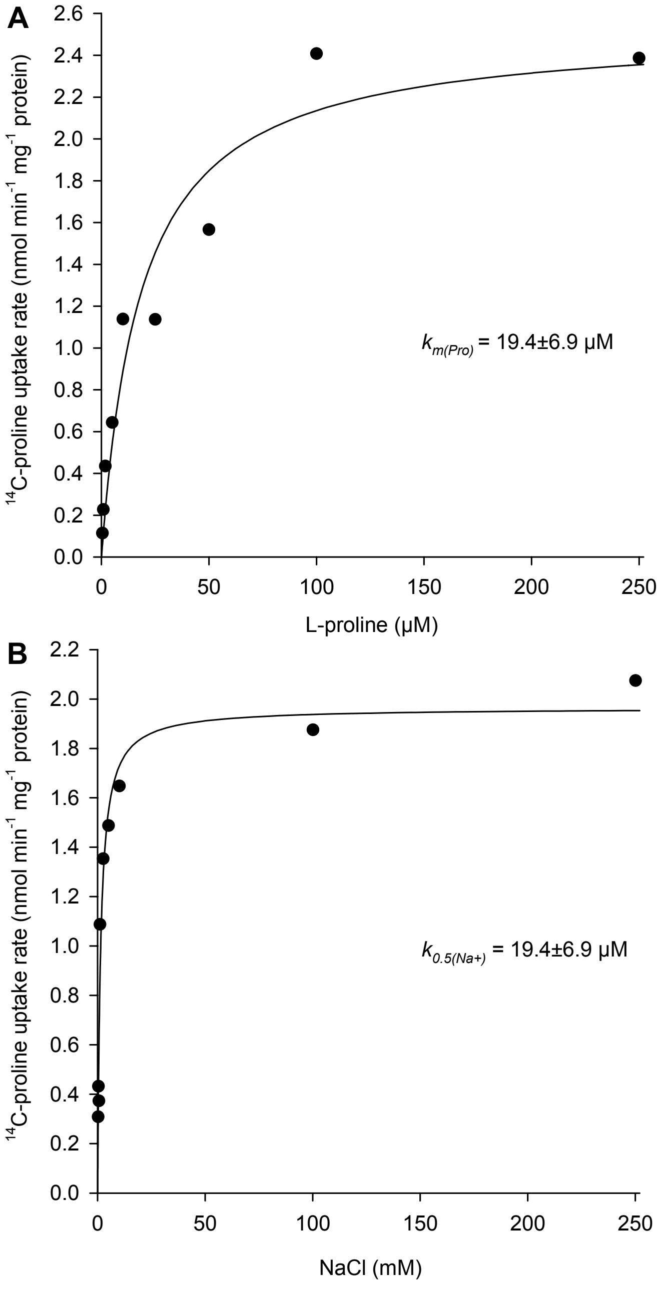

Figure S4. Kinetics of ^{14}C-L-proline uptake into *H. pylori*. Cells were grown in Brucella broth under microaerophilic conditions as described in *Experimental Procedures.* For the transport assay, 200 µl aliquots of a cell suspension (OD_{600}=0.8 in 100 mM Tris/MES, pH 7.0/150 mM KCl) were prepared per time point. Initial rates of ^{14}C-L-proline uptake were determined *(A)* at ^{14}C-L-proline concentrations varying from 0.5 µM to 250 µM in the presence of 50 mM NaCl, and *(B)* at a constant ^{14}C-L-proline concentration of 10 µM and NaCl added at concentrations varying from 70 µM to 250 mM using the rapid filtration assay. Data points represent the mean of duplicate determinations of a representative experiment. Three repeats of the experiment with independently grown and treated cells yielded similar $k_{m(Pro)}$ and $k_{o.5(Na+)}$ values with maximum activities varying by a factor of up to three between the individual experiments.

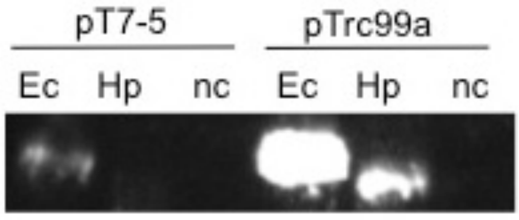

Figure S5. Comparison of the amounts of EcPutP and HpPutP in membranes of *E. coli* WG170. Expression of *EcputP* (Ec) and *HpputP* (Hp) was achieved form the promoters *lac* (plasmids pT*HpputPF6H*, pT*EcputPF6H*) and *trc* (plasmids pR*HpputPF6H*, pR*EcputPF6H*). Plasmids pT7-5 and pTrc99a served as negative controls (nc). Relative amounts of the transporters were estimated by Western-Blot analysis with HRP-linked anti-FLAG IgG directed against the FLAG epitope at the C termini of EcPutP and HpPutP.

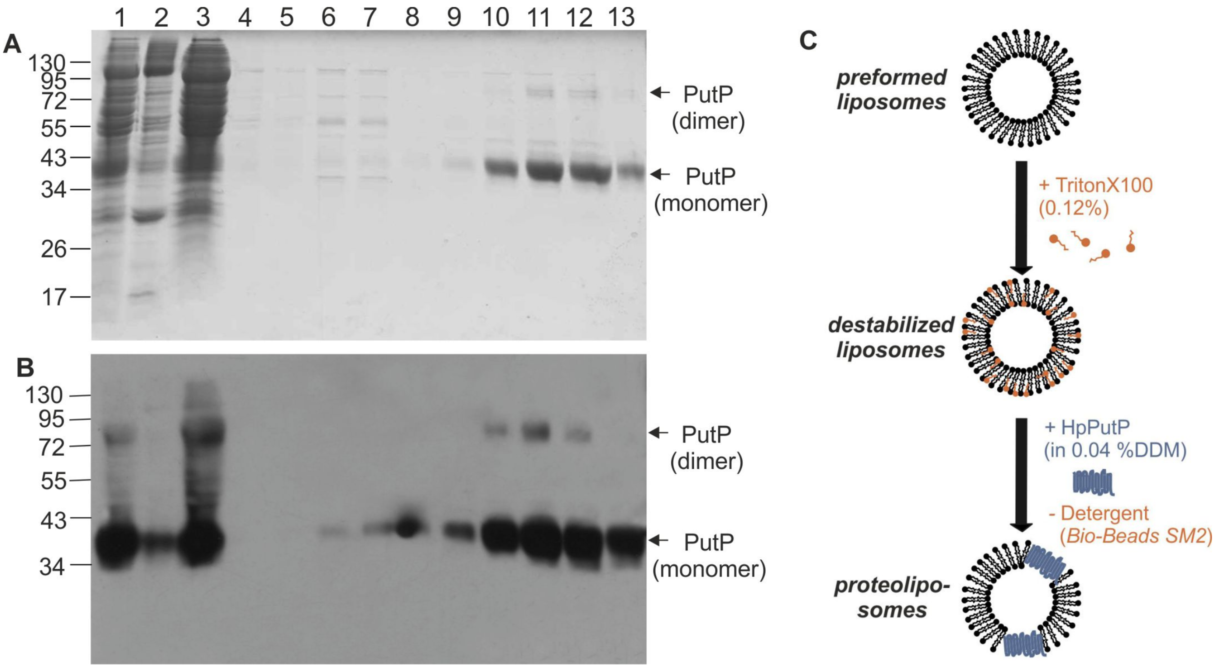

Figure S6. Purification and reconstitution of HpPutP. Cells of *E. coli* WG170 transformed with plasmid pR*HpputP6H* were grown and membranes were prepared as described in *Experimental Procedures.* The protein (5 mg ml^{-1} total membrane protein) was solubilized with 1.5 % n-dodecyl-ß-D-maltopyranoside (DDM) and purified by Ni^{2+}-NTA affinity chromatography. Out of 45 mg total membrane protein applied to 1 ml Ni^{2+}-NTA agarose about 1 mg of HpPutP with a purity of about 95 % was obtained. *(A)* SDS-PAGE and Coomassie stain, *(B)* Western Blot of individual steps of the purification procedure. *(1)* solubilisate; *(2)* membrane fraction after solubilization; *(3)* flow through of Ni^{2+}-NTA agarose column; *(4-8)* fractions of column washing steps; *(9-13)* fractions containing HpPutP eluted with 200 mM imidazole. *(C)* Scheme of the reconstitution procedure. Liposomes were preformed from an *E. coli* polar lipid extract, detergent destabilized, incubated with purified HpPutP at a lipid to protein ratio of 100 to 1 (w/w), and proteoliposomes were formed by stepwise removal of the detergent with Bio-Beads SM2.

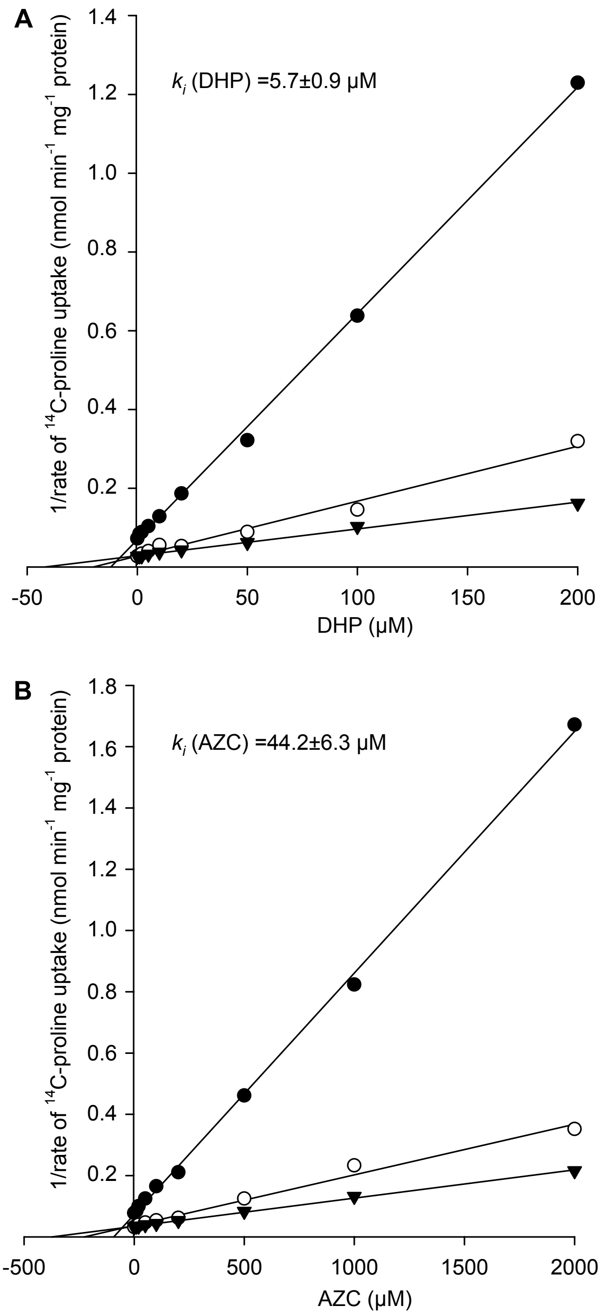

Figure S7. Dixon plot analysis of the inhibition of ^{14}C-L-proline uptake by *(A)* 3,4-dehydro-D,L-proline (DHP) and *(B)* L-azetidine-2-carboxylic acid (AZC). Uptake of ^{14}C-L-proline (26 Ci mol^{-1}) into *E. coli* WG170 harboring HpPutP was assayed in the presence of 50 mM NaCl and 20 mM D-lactate (Na^+-salt) at the indicated inhibitor concentrations at 25°C. The following ^{14}C-L-proline concentrations were used: 1 µM (closed triangle); 5 µM (open circles); 10 µM (closed circles). Presented is a linear regression of the data sets. The data points represent the average of two parallel measurements. Two repeats of the experiment with independently grown and treated cells yielded identical k_i values. K_i values were determined with the enzyme kinetics module of *SigmaPlot*.

1. Sievers F, Wilm A, Dineen D, Gibson TJ, Karplus K, et al. (2011) Fast, scalable generation of high-quality protein multiple sequence alignments using Clustal Omega. Mol Syst Biol 7: 539.
2. Faham S, Watanabe A, Besserer GM, Cascio D, Specht A, et al. (2008) The crystal structure of a sodium galactose transporter reveals mechanistic insights into Na^{+}/sugar symport. Science 321: 810-814.
3. Krogh A, Larsson B, von Heijne G, Sonnhammer EL (2001) Predicting transmembrane protein topology with a hidden Markov model: application to complete genomes. J Mol Biol 305: 567-580.
